# Investigation of frailty markers including a novel biomarker panel in emergency laparotomy: protocol of a prospective cohort study

**DOI:** 10.1186/s12893-023-02093-5

**Published:** 2023-07-05

**Authors:** Hwei Jene Ng, Tara Quasim, Nicholas J. W. Rattray, Susan Moug

**Affiliations:** 1 School of Medical, Veterinary & Life Sciences (MVLS), University of Glasgow, Glasgow, UK; 2 Department of General Surgery, Royal Alexandra Hospital, NHS Greater Glasgow and Clyde, Corsebar Road, Paisley, PA2 9PN UK; 3 Department of Intensive Care Medicine, Glasgow Royal Infirmary, NHS Greater Glasgow and Clyde, Glasgow, UK; 4 Academic Unit of Anaesthesia, Critical Care and Peri- operative Medicine, University of Glasgow, Glasgow, UK; 5 Strathclyde Institute of Pharmacy & Biomedical Sciences, University of Strathclyde, Glasgow, G4 0RE UK; 6 Strathclyde Centre for Molecular Bioscience, University of Strathclyde, Glasgow, G4 0RE UK; 7 School of Medicine, Dentistry & Nursing, University of Glasgow, Glasgow, United Kingdom; 8 Department of Colorectal Surgery, Golden Jubilee University National Hospital, Glasgow, United Kingdom

**Keywords:** Emergency laparotomy, Frailty biomarkers, Mass spectrometry, Clinical Frailty Scale, Sarcopenia

## Abstract

**Background:**

Emergency laparotomy (EmLAP) is one of the commonest emergency operations performed in the United Kingdom (approximately 30, 000 laparotomies annually). These potentially high-risk procedures can be life changing with frail patients and/ or older adults (≥ 65 years) having the poorest outcomes, including mortality. There is no gold standard of frailty assessment and no clinical chemical biomarkers existing in practice. Early detection of subclinical changes or deficits at the molecular level are essential in improving our understanding of the biology of frailty and ultimately improving patient outcomes. This study aims primarily to compare preoperative frailty markers, including a blood-based biomarker panel, in their ability to predict 30 and 90-day mortality post-EmLAP. The secondary aim is to analyse the influence of perioperative frailty on morbidity and quality of life post-EmLAP.

**Methods:**

A prospective single centred observational study will be conducted on 150 patients ≥ 40 years of age that undergo EmLAP. Patients will be included according to the established NELA (National Emergency Laparotomy Audit) criteria. The variables collected include demographics, co-morbidities, polypharmacy, place of residence, indication and type of surgery (as per NELA criteria) and prognostic NELA score. Frailty will be assessed using: a blood sample for ultra-high performance liquid chromatography mass spectrometry analysis; preoperative CT abdomen pelvis (sarcopenia) and Rockwood Clinical Frailty Scale (CFS). Patients will be followed up for 90 days. Variables collected include blood samples (at post operative day 1, 7, 30 and 90), place of residence on discharge, morbidity, mortality and quality of life (EQ-5D-5 L). The frailty markers will be compared between groups of frail (CFS ≥ 4) and non-frail using statistical methods such as regression model and adjusted for appropriate confounding factors.

**Discussion:**

This study hypothesises that frailty level changes following EmLAP in frail and non- frail patients, irrespective of age. We propose that non- frail patients will have better survival rates and report better quality of life compared to the frail. By studying the changes in metabolites/ biomarkers in these patients and correlate them to frailty status pre-surgery, this highly novel approach will develop new knowledge of frailty and define a new area of clinical biomolecular research.

**Trial registration:**

ClinicalTrials.gov: NCT05416047. Registered on 13/06/2022 (retrospectively registered).

**Supplementary Information:**

The online version contains supplementary material available at 10.1186/s12893-023-02093-5.

## Background

Emergency laparotomy (EmLAP) is one of the commonest emergency operations performed in the United Kingdom with approximately 30,000 laparotomies performed annually in England and Wales [1]. Frail patients have been shown to have significantly poorer outcomes, especially in older adults (≥ 65 years) [2, 3]. According to the recent 8th patient report of National Emergency Laparotomy Audit (NELA), more than 22,000 patients underwent emergency laparotomy in a year and more than half (55.3%) of these patients were ≥ 65 years. The report found that risk of mortality doubled among frail patients undergoing EmLAP [4]. These findings were mirrored in the first multi-centred observational study, the Emergency Laparotomy Study (ELF study) [5]. Using the Rockwood Clinical Frailty Scale (CFS), the ELF study reported 20% of older adults had frailty pre-EmLAP. As frailty increased, independent of age, 30-day 90-day morality did too. Furthermore, the presence of frailty resulted in an increased level of dependence at discharge highlighting the significant and prolonged health demands of frailty.

Frailty is a clinical syndrome of increased vulnerability that results from ageing associated multi-system physiological decline [6]. Such a decline reduces the ability of the individual to cope with physiological stress such as surgery (surgical frailty) and negatively impact on their quality of life. Frailty can be identified using different questionnaires and assessments depending on what aspect of the syndrome is being looked at. This includes but not limited to muscle wasting (sarcopenia), malnutrition, cognition (memory, orientation and processing), physical function and capacity [7–10]. Most studies associate frailty with the older population, but frailty has also been shown to exist in younger individuals [11]. Moreover, there could be a time gap between biological changes and phenotypic manifestations of frailty (pre- frail) [12]. To date, there isn’t a frailty assessment tool that has been identified as the gold standard for assessing frailty in all clinical situations including emergency setting. Hence, an objective measurement of frailty is required to not only identify and modify frailty, but in understanding the biology of ageing that leads to a clinical diagnosis of frailty.

One of the largest research studies on role of metabolomics in frailty [13] demonstrated how mass spectrometry can be used to understand changes in blood metabolism across frailty. Serum samples from 1200 people from within the English Longitudinal Study of Ageing (ELSA- www.elsa-project.ac.uk) were chemically profiled and mapped on to associated Rockwood Frailty Index calculations and altered levels of 12 metabolites were found to be dysregulated in those that were consider frail. These included 3 tocotrienols and 6 carnitines- metabolites known to be directly involved in energy metabolism, which was used to differentiate between frail and non-frail patients. These findings indicated that decreased energy production acts as a common pathway and a primary driver toward frailty which was reflected globally by frail individuals presenting symptoms of weakness, slowness, exhaustion and low activity. These results provided a platform for this study to establish a panel of novel frailty biomarkers in emergency laparotomy patients and correlate blood metabolites with changes in frailty due to EmLAP, postoperative morbidity and mortality.

No clinical chemical biomarkers for frailty currently exist in practice due to a lacking in the understanding of the biochemical aetiology of frailty. However, early detection of subclinical changes or deficits at the molecular level are essential in improving our understanding of the biology of frailty and ultimately improving patient outcomes, aiding in shared decision-making, precision medicine and development of new optimal postoperative EmLAP patient pathway.

The primary aim of this study is to compare preoperative frailty markers including establishing a novel blood-based biomarker panel that could identify frailty in EmLAP patients and their ability to predict 30 and 90-day mortality post-EmLAP. The secondary aim is to analyse the influence of perioperative frailty on 30-day morbidity and quality of life post-EmLAP.

## Methods/ designs

### Patient recruitment

**Fig. 1:**
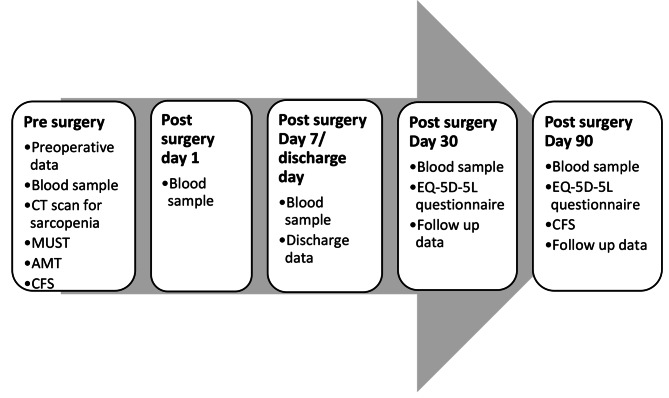
The data collected after obtaining consent including follow up period. MUST: Malnutrition Universal Screening Tool; AMT: Abbreviated Mental Test; CFS: Rockwood Clinical Frailty Score

A prospective single centred observational study will be conducted in a National Health Service hospital that has an acute emergency surgical service. All consecutive emergency laparotomy (EmLAP) patients ≥ 40 years of age that undergo EmLAP will be screened and recruited. For patients who were deemed to have a lack of capacity to consent, their next of kin or welfare attorney will be approached to obtain written consent for the study pre-operatively. When the participant regains capacity, they will be approached to re-obtain consent to continue participation. Patients who do not regain capacity, the consent from next of kin or welfare attorney will continue. All recruited patients will be followed up for 90 days. The data collected after gaining consent and up to 90 days follow up are summarised in Fig. 1. Participant’s details will be pseudo-anonymised with a study ID.

### Inclusion criteria

1. Age ≥ 40 years.
2. English and non- English speaker (usage of language line to obtain consent).
3. NELA inclusion criteria [4].

### Exclusion criteria

1. Under 40 years of age.
2. CT scan or postoperative finding of inoperable disseminated peritoneal disease.
3. Open and close laparotomy (postoperative palliation, non-survivable global ischemia where there are < 90 cm from duodenojejunal junction to stoma).
4. Complication from colonic stenting requiring laparotomy.
5. NELA exclusion criteria [4].

### Data collection

The majority of data will be obtained from the routine admission documentations and electronic patient record and recorded in an online password protected spreadsheet. The variables recorded will include:

1) Demographics: Age, gender, deprivation score/ Scottish Index Multiple Deprivation (SIMD) [14] score (1 to 5), ethnic origin.

2) Co-morbidities: Charlson co- morbidity index (number of co-morbidities < 5 or ≥ 5) [15].

3) Polypharmacy (> 5 regular medications).

4) Lifestyle: alcohol intake (unit per week), smoking status (currently, ex-smoker < 5 year, ex-smoker ≥ 5 years, never smoked), weight and Body Mass Index.

5) Place of residence: home and independent, home with carers, home with family support, sheltered housing, nursing home.

6) Indication for and type of surgery: as per NELA criteria [4].

7) Prognostic scores: NELA score.

8) ASA (American Society of Anaesthesiology) score [16].

Frailty markers (pre-operative):

1) Plasma samples obtained using 6ml lithium heparin tubes for ultra-high performance liquid chromatography mass spectrometry analysis (UHPLC-MS).

2) Sarcopenia on CT abdomen pelvis [17].

3) Malnutrition Universal Screening Tool (MUST) score.

4) Routine Abbreviated Mental Test (AMT) score.

5) Routine serum biochemistry and haematology: Hb, White cells, neutrophils, C- Reactive protein, Albumin.

6) Rockwood Clinical Frailty Scale (CFS; assessing the baseline frailty status 2 weeks before admission with scores from 1 to 9 as frailty increases). Score of ≥ 4 will be grouped as frail [8].

Follow up variables recorded are:

1) Day 1, day 7 or discharge day if before day 7, day 30 and day 90 blood samples.

2) Total hospital stay including critical care stay.

3) Place of residence post discharge following emergency laparotomy.

4) Day 30 readmission.

5) Day 30 morbidity/ complications (according Clavien-Dindo classification Surgical Complications) [18].

6) Day 30 and day 90 mortality.

7) EQ-5D-5L on day 30 and 90.

8) Day 90 CFS.

### Assessment of Sarcopenia

Participants of this study who underwent CT abdomen pelvis pre- operatively will be measured for sarcopenia [17]. The measurement of total cross-sectional area of psoas muscle (total psoas area, TPA) on L3 vertebra level will be performed using PACS (Picture Archiving and Communication System software). The area measured is standardised by measuring at the level where both L3 transverse processes were maximally in view on CT. The TPA is standardised for patient height using formula of TPA (mm^2^)/ height (m^2^) providing the total psoas index (TPI). The TPI will be stratified according to gender with sarcopenia being present in the lowest quartile [19].

Calculations of inter and intra- class correlation coefficients (ICCC) will be performed to ensure reliability of the measurement technique. The inter and intra ICCC will be calculated where the r^2^ value of > 0.8 indicates excellent correlation and ICCC value close to 1 indicates high similarity and high reliability.

### Management of blood samples

Blood samples will be obtained at 5 time points (preoperatively, postoperative day 1, 7 or discharge day if before day 7, day 30 and day 90) using 6mL lithium heparin vacutainers. The sample will be centrifuged to have plasma extracted and stored at -40 °C. These will then be sent on for ultra-high performance liquid chromatography mass spectrometry analysis (UHPLC-MS) in Strathclyde Institute of Pharmacy & Biomedical Sciences, University of Strathclyde for analysis.

### Ultra-high Performance Liquid Chromatography mass spectrometry analysis

A semi-targeted ultra-high performance liquid chromatography mass spectrometry-based metabolomics approach (UHPLC-MS) will be performed to determine metabolic signatures correlated to frailty status and surgical outcome. This will be performed on a Thermo Exploris 240 mass spectrometer coupled to a Thermo Vanquish binary liquid chromatography instrument. The applied method will contain a range of predefined targeted mass measurements (using product ion multiple reaction monitoring) related to a defined group of biochemically annotated metabolites already identified in previous research [13] which include 13-carboxy-alpha-tocotrienol, stearoylcarnitine, vaccenyl carnitine, urocortisol, 13-carboxy gamma-tocotrienol, hexacosonyl carnitine, formyl-N-acetyl-5-methoxykynurenamine, arginine, gamma-tocotrienol, linoleyl carnitine, arachidonic acid and prostaglandin G2. A range of other chemical classes will also be targeted that will cover aspects of dietary response and polypharmacy – determined by pre-operative information provided by the patient cohort.

### Assessment of postoperative quality of life

The EQ-5D-5L questionnaire is a validated measurement tool which evaluates 5 dimensions of quality of life including mobility, self-care, activity of daily living, pain/ discomfort and psychological status [20]. This questionnaire will be used to assess the participant’s quality of life at postoperative day 30 and 90.

### Sample size

This is an exploratory study comparing different frailty markers in emergency laparotomy therefore no formal sample size calculations were performed. Historically, the centre where the participants are recruited from performed about 200 EmLAP per year. Based on the UK Observational Emergency Laparotomy and Frailty (ELF) study [5], 20% of older patients undergoing laparotomy had frailty pre- EmLAP. For this study, 20% of dropout/ loss to follow up/ withdrawal from study was anticipated [21] therefore a target total of 150 patients was established.

### Statistical analysis

The categorical variables (demographics, co-morbidities, polypharmacy, ASA score) will be expressed as n and percentage (%) and continuous variables (age) as mean ± standard deviation. The frailty markers (score 1–9) will be compared between groups of frail (CFS ≥ 4) and non-frail (CFS ≤ 3). Categorical variables will be analysed using Chi-square or Fisher Exact test. All normally distributed continuous variables will be assessed with Student’s T-test or the ANOVA test and for the non- normal distribution variables, the rank based Kruskal- Wallis non-parametric test will be used to determine the statistical significance between the different groups.

The primary analysis of metabolomics data will involve a range of pre-processing normalisation procedures to help scale the differences in the magnitude of metabolite concentrations. Normalised features will then be compared for analytical reproducibility by using multivariate principal component analysis (PCA) to determine QC clustering and outlier identification. Further analysis of clinical frailty markers and between groups of frail and non-frail (defined by clinical frailty scale binning) using the Kruskal-Wallis t-test or Mann Whitney test and subsequently in a regression model and propensity score matching, adjusting for appropriate demographics and other baseline variables. A linear mixed model will also be used on the normalised data to study the changes of each metabolite over time to identify differences between frailty status, response post-surgery and polypharmacy interactions.

The association between frailty markers and mortality will be analysed using regression model and will be adjusted for confounding factors such as presence of sepsis and type of surgery. Sensitivity and specificity analyses will be performed using the Receiver Operating Characteristic curve (ROC). A p-value of < 0.05 is considered statistically significant.

Result from EQ-5D-5L questionnaire will be reported and analysed quantitatively (means and ranges of 5 domains) using the analysis tool (index value set calculator and analysis of observational descriptive systems data) provided by the EuroQol Office [22].

## Discussion

This study hypothesises that patient’s assessed frailty changes following EmLAP in frail and non- frail patients, irrespective of age. We propose that non- frail patients will have better survival rates and recover faster compared to the frail. Underpinning this, the study aims to measure changes in metabolites/ biomarkers in these patients and correlate them to frailty status pre-surgery. This highly novel approach will develop new knowledge of frailty in two ways. Firstly, by measuring how metabolite levels change throughout the surgical process, we can map these changes on to canonical biomolecular pathways that directly translate to clinical observation. There is currently no knowledge of the under pinning biochemical changes of frailty in the surgical setting as well as the changes in frailty during perioperative period. Thus, this study would define a new area of clinical biomolecular research. Secondly, confirmed dysregulated molecular features can be developed into a predictive biomarker panel used to assess fitness of a patient from the stresses of surgery.

### Trial status

Protocol version number 1.6 07/05/2022.

Date recruitment began- 28/05/2022.

Completion date of recruitment (approximate)- 01/08/2023.

## Electronic supplementary material

Below is the link to the electronic supplementary material.

Additional File 1: NELA inclusion and exclusion criteria

## Data Availability

The datasets from the current study can be obtained from the corresponding author at the end of the study on reasonable request.

## Abbreviations

AMT: Abbreviated Mental Test
ASA score: American Society of Anaesthesiology score
CFS: Rockwood Clinical Frailty Scale
EmLAP: Emergency laparotomy
ICCC: inter and intra- class correlation coefficients
MUST score: Malnutrition Universal Screening Tool score
NELA: National Emergency Laparotomy Audit
PACS: Picture Archiving and Communication System software
TPA: Total psoas area
TPI: Total psoas index
UHPLC-MS: ultra-high performance liquid chromatography mass spectrometry
