# Supplementary material for: Investigation of frailty markers including a novel biomarker panel in emergency laparotomy: protocol of a prospective cohort study
Source: BMC Surg. 2023 Jul 5;23:190. doi: 10.1186/s12893-023-02093-5 (PMC10321009; doi:10.1186/s12893-023-02093-5)
Supplement: Supplementary file 1 — Additional File 1: NELA inclusion and exclusion criteria [file 12893_2023_2093_MOESM1_ESM.docx]

**NELA inclusion and exclusion criteria**

NELA inclusion criteria

- Open, laparoscopic, or laparoscopically-assisted procedures

- Procedures involving the stomach, small or large bowel, or rectum for conditions such as perforation, ischaemia, abdominal abscess, bleeding or obstruction

- Washout/evacuation of intra-peritoneal abscess (unless due to appendicitis or cholecystitis – excluded, see below)

- Washout/evacuation of intra-peritoneal haematoma

- Bowel resection/repair due to incarcerated incisional, umbilical, inguinal and femoral hernias (but not hernia repair without bowel resection/repair). E.g. Large incisional hernia repair with bowel resection

Bowel resection/repair due to obstructing/ incarcerated incisional hernias provided the presentation and findings were acute. This will include large incisional hernia repair with division of adhesions.

- Laparotomy/laparoscopy with inoperable pathology (e.g. peritoneal/hepatic metastases) where the intention was to perform a definitive procedure. This does not include purely diagnostic procedures.

- Laparoscopic/Open Adhesiolysis

- Return to theatre for repair of substantial dehiscence of major abdominal wound (i.e. “burst abdomen”)

- Any reoperation/return to theatre for complications of elective general/upper GI surgery meeting the criteria above is included. Returns to theatre for complications following non-GI surgery are now excluded (see exclusion criteria below).

NELA inclusion criteria

- Elective laparotomy / laparoscopy

- Diagnostic laparotomy/laparoscopy where no subsequent procedure is performed (NB, if no procedure is performed because of inoperable pathology, then include)

- Appendicectomy +/- drainage of localised collection unless the procedure is incidental to a non-elective procedure on the GI tract

- Cholecystectomy +/- drainage of localised collection unless the procedure is incidental to a non-elective procedure on the GI tract (All surgery involving the appendix or gallbladder, including any surgery relating to complications such as abscess or bile leak is excluded. The only exception to this is if carried out as an incidental procedure to a more major procedure. We acknowledge that there might be extreme cases of peritoneal contamination, but total exclusion avoids subjective judgement calls about severity of contamination.)

- Non-elective hernia repair without bowel resection or division of adhesions

- Minor abdominal wound dehiscence unless this causes bowel complications requiring resection.
- Non-elective formation of a colostomy or ileostomy as either a trephine or a laparoscopic procedure (NB: if a midline laparotomy is performed, with the primary procedure being formation of a stoma then this should be included)

- Vascular surgery, including abdominal aortic aneurysm repair

- Caesarean section or obstetric laparotomies

- Gynaecological laparotomy

- Ruptured ectopic pregnancy, or pelvic abscesses due to pelvic inflammatory disease

- Laparotomy/laparoscopy for pathology caused by blunt or penetrating trauma

- All surgery relating to organ transplantation (including returns to theatre for any reason following transplant surgery)

- Surgery relating to sclerosing peritonitis

- Surgery for removal of dialysis catheters

- Laparotomy/laparoscopy for oesophageal pathology

- Laparotomy/laparoscopy for pathology of the spleen, renal tract, kidneys, liver, gall bladder and biliary tree, pancreas or urinary tract

- Returns to theatre for complications (eg bowel injury, haematoma, collection) following non-GI surgery are now excluded. i.e returns to theatre following renal, urological, gynaecological, vascular, hepatic, pancreatic, splenic surgery are excluded
